# Emotion regulation deficits in euthymic bipolar I versus bipolar II disorder: a functional and diffusion-tensor imaging study

**DOI:** 10.1111/bdi.12292

**Published:** 2015-03-15

**Authors:** Xavier Caseras, Kevin Murphy, Natalia S Lawrence, Paola Fuentes-Claramonte, Jessica Watts, Derek K Jones, Mary L Phillips

**Affiliations:** a MRC Centre for Neuropsychiatric Genetics and Genomics, Institute of Psychological Medicine and Clinical Neurosciences, Cardiff UniversityCardiff, UK; b Neuroscience and Mental Health Research Institute, Cardiff UniversityCardiff, UK; c Cardiff University Brain Research Imaging Centre, School of Psychology, Cardiff UniversityCardiff, UK; d Mood Disorders Centre, School of Psychology, University of ExeterExeter, UK; e Departament de Psicologia Bàsica, Clínica i Psicobiologia, School of Psychology, Universitat Jaume I de CastellóCastello, Spain; f Mood and Brain Laboratory, Department of Psychiatry, University of Pittsburgh School of Medicine, Western Psychiatric Institute and Clinic, University of PittsburghPittsburgh, PA, USA

**Keywords:** bipolar disorder, BOLD, DTI, emotion regulation, fMRI

## Abstract

**Objectives:**

Emotion regulation deficits are a core feature of bipolar disorder. However, their potential neurobiological underpinnings and existence beyond bipolar I disorder remain unexplored. Our main goal was to investigate whether both individuals with bipolar I and bipolar II disorder show deficits in emotion regulation during an attention control task, and to explore the neurophysiological underpinnings of this potential deficit.

**Methods:**

Twenty healthy controls, 16 euthymic participants with bipolar I disorder, and 19 euthymic participants with bipolar II disorder completed psychometric and clinical assessments, a neuroimaging emotion regulation paradigm, and an anatomical diffusion-weighted scan. Groups were matched for age, gender, and verbal IQ.

**Results:**

During the presence of emotional distracters, subjects with bipolar I disorder showed slowed reaction times to targets, and increased blood oxygenation level-dependent (BOLD) responses in the amygdala, accumbens, and dorsolateral prefrontal cortex, but not increased inverse functional connectivity between these prefrontal and subcortical areas, and altered white matter microstructure organization in the right uncinate fasciculus. Subjects with bipolar II disorder showed no altered reaction times, increased BOLD responses in the same brain areas, increased inverse functional connectivity between the prefrontal cortex and amygdala, and no abnormalities in white matter organization.

**Conclusions:**

Participants with bipolar I disorder showed abnormalities in functional and anatomical connectivity between prefrontal cortices and subcortical structures in emotion regulation circuitry. However, these deficits did not extend to subjects with bipolar II disorder, suggesting fundamental differences in the pathophysiology of bipolar disorder subtypes.

Emotion regulation deficits are at the core of bipolar disorder (BD) 1–5 and persist during remission 3, constituting potential trait markers. Among the cognitive processes required to regulate emotions, attentional control is key 6, and this is impaired in BD 4. Euthymic individuals with BD, compared to healthy controls (HC), show greater activity in the dorsolateral prefrontal cortex (DLPFC) while undertaking emotion regulation 7. The DLPFC downregulates subcortical structures during voluntary emotion regulation 4. Furthermore, deficits in prefrontal–amygdala functional connectivity during emotion regulation have been reported in BD 7–10. Together, findings suggest increased activity in emotion processing areas 11, but abnormally elevated activity and decreased functional connectivity within the emotion regulation circuitry in individuals with BD.

In line with these functional magnetic resonance imaging (fMRI) findings, diffusion tensor imaging (DTI) studies have consistently reported white matter abnormalities in the uncinate fasciculi in BD 12–15. The uncinate fasciculus is considered central to emotion regulation as it connects prefrontal and anterior temporal cortices 16. It could play a major role in the downregulation of activity in subcortical structures like the amygdala by the DLPFC.

The majority of previous research, however, has been restricted to individuals with BD type I (BD-I). Until recently, BD type II (BD-II) was regarded as simply a ‘softer’ form of BD-I, therefore assuming that the same neurobiological deficits applied to both types of BD, with only a potential difference in the magnitude of these effects. However, this view has been challenged recently from a clinical and neuroscientific perspective. Clinical research shows BD-II to present a course of illness and associated health problems at least as severe as in BD-I 17,18, and neuroimaging studies have reported important differences in neural anatomy and function between BD-I and BD-II 19–21.

The aim of the present study was to further investigate potential differences between euthymic BD-I and BD-II, relative to a group of HC, in function (activity and connectivity) and white matter microstructure within emotion regulation circuitry. We employed a validated emotion regulation paradigm – a modified verbal n-back task including emotional distracters – shown to induce activity in DLPFC and subcortical regions 7,22, and obtained indices of white matter microstructure organization in the uncinate fasciculi, as revealed by diffusion MRI.

Due to the absence of previous literature on which to base our hypothesis, we tested the null hypothesis of no differences between BD-I and BD-II. However, based on previous research on BD-I, we hypothesized that, relative to HC, both groups would show: (i) an increased reaction time to the target stimuli due to the presence of emotional distracters (more notably, during the higher memory load condition); (ii) increased activity in the DLPFC due to the presence of emotional distracters; (iii) increased activity in the amygdala and nucleus accumbens during conditions including negative and positive distracters; (iv) reduced functional connectivity between the DLPFC and amygdala/accumbens in the presence of emotional distracters; and (v) altered white matter microstructural organization in the uncinate fasciculi.

## Methods

### Participants and questionnaires

Participants with BD were recruited from a pre-existing database of well-characterized patients participating in ongoing genetic studies at Cardiff University (Cardiff, UK). HC were recruited from the community via advertisement.

Volunteers free from any MRI contraindications were recruited. The Mini International Neuropsychiatric Interview (MINI) 23 was used to confirm diagnosis and exclude subjects with BD with a history of psychotic (other than during mood episodes) or borderline personality disorders. Exclusion criteria for HC included a family history of psychotic or affective disorders and a personal history of mental disorders. A recent history (<1 year ago) of alcohol or substance abuse/dependence was also an exclusion criterion for all participants in the study; however, only one patient with BD-I had a previous (>1 year) history of alcohol dependence. Euthymia was defined as the absence of any episode of depression or hypo/mania for two months before scanning, based on clinical interview, along with unchanged drug treatment for the same period, plus current scores <10 on the Hamilton Rating Scale for Depression (HAM-D) 24 and the Young Mania Rating Scale (YMRS) 25. Participants also completed the National Adult Reading Test (NART) 26 to estimate premorbid verbal IQ.

Two participants with BD-I were excluded following uncertain euthymic status at scanning, bringing the final sample to 20 HC, 16 BD-I, and 19 BD-II. Of those, two patients with BD-I were excluded from DTI analyses owing to poor data quality, and one HC from the behavioral and fMRI analyses owing to poor understanding of the task.

Participants gave written informed consent and received £20. The study was approved by the local National Health Service-Research Ethics Committee.

## Experimental procedures

We employed a validated emotion regulation paradigm 7,22 where participants perform a mixed event–block design verbal n-back task including the 0-back and 2-back conditions. For some blocks, the targets (letters) appear flanked by two identical emotional facial expressions. Participants were instructed to ignore these faces and concentrate on the letters. The task included eight conditions resulting from all the possible combinations of memory load (0-back versus 2-back) × distracter (no-distracter, neutral, fear, happy). Three runs, each including one block of all eight conditions, were presented (see *Supplementary material*). Participants were fully trained prior to scanning.

### Image acquisition and data analysis

Participants performed the above task during optimized fMRI data acquisition. An anatomical three-dimensional fast spoiled gradient-echo (FSPGR) scan for fMRI data co-registration and an optimized DTI scan were also acquired (see *Supplementary material*).

fMRI data were analyzed using the Functional MRI of the Brain (FMRIB) Software Library (www.fmrib.ox.ac.uk/fsl). Pre-processing followed standard methods (See *Supplementary material*). The task was modeled within the general linear model (GLM) framework, with only correct trials (i.e., target letters with button press and non-target letters with no response) included. Incorrect trials were modeled within a regressor of non-interest, along with instruction periods. The resulting model included seven regressors, one for each possible condition, except the baseline (0-back + no-distracters). The loss of degrees of freedom caused by inspecting all potential main effects and interactions was minimized by limiting our analyses to *a priori*-defined contrast sets that investigated the main effects of working memory and distracters (See *Supplementary material*). The resulting functional images were transformed linearly to standard Montreal Neurological Institute space using FMRIB Linear Image Registration (FLIRT) 27. A second-level analysis was conducted using ordinary least squares to add the three runs together, and a third-level analysis to compare the resulting functional images between groups. As our hypotheses were strongly focused on the DLPFC, amygdala, and accumbens, regions of interest (ROIs) were drawn within these three regions (See *Supplementary material*). In order to investigate the strength of the inverse functional connectivity between the DLPFC and amygdala/accumbens, the time series from the DLPFC was entered into a GLM to run a psychophysiological interaction (PPI) analysis 28 constrained to the amygdala and accumbens. The interaction between the DLPFC time series and each of the regressors corresponding to the presence of target letters + fearful distracters, target letters + happy distracters, and targets + neutral distracters was separately created and compared across groups. The 3DClustSim program within AFNI with a threshold of p = 0.001 was used to determine the minimum cluster size associated with a corrected p < 0.05 within each ROI, which was set at nine voxels for the amygdala, 13 for the accumbens, and 26 for the DLPFC.

DTI data were analyzed using ExploreDTI 29 using standard procedures (See *Supplementary material*). We used a tractography approach in which the uncinate fasciculi (tract of interest) and the lower section of the cortical–spinal tract (comparison tract) were reconstructed separately for each hemisphere, and diffusion tensor MRI-derived indices for white matter microstructural organization were extracted and compared between groups using analysis of variance (ANOVA).

Behavioral responses, and demographic and clinical measures were compared between groups using ANOVA or chi-square tests, as appropriate.

## Results

### Demographics and clinical measures

Gender, age, and NART score were evenly distributed across groups. As expected, the HAM-D and YMRS scores for both BD groups were higher than for HC (Table1), although were still low and well under our set threshold for euthymia.

**Table 1:** Distribution of demographic and clinical variables across groups

	HC (n = 20)	BD-I (n = 16)	BD-II (n = 19)		
	Mean (SD)	Mean (SD)	Mean (SD)	Group comparison	p-value
Age	42.30 (5.99)	42.56 (7.47)	38.74 (8.07)	*F*(2,54) = 1.63	> 0.10
NART-correct	35.56 (8.41)	35.06 (8.29)	32.60 (7.51)	*F*(2,46) = 0.58	> 0.10
HAM-D score	0.55 (0.82)^a^	3.44 (3.52)	2.67 (2.94)	*F*(2,50) = 6.10	< 0.005
YMRS score	0.65 (0.93)^a^	3.13 (2.39)	1.80 (2.80)	*F*(2,50) = 6.14	< 0.005
Age at first mood episode, years	–	19.07 (7.60)	17.94 (7.21)	*t*(30) = 0.42	>0.10
Age at BD diagnosis, years	–	27.81 (6.89)	30.79 (10.14)	*t*(33) = 0.99	> 0.10
Symptoms during ‘high’	–	23.73 (5.99)	23.13 (4.82)	*t*(27) = 0.95	> 0.10
	N (%)	N (%)	N (%)		
Gender, female	13 (65)	10 (62)	13 (68)	*c*^2^ (2, n = 55) = 0.13	>0.10
Family history of BD	–	7 (50)	5 (31)	*c*^2^ (1, n = 30) = 1.09	> 0.10
Comorbid anxiety	–	5 (31)	6 (31)	*c*^2^ (1, n = 35) = 0.00	> 0.10
Medications					
Mood stabilizers	–	11 (68)	9 (47)	*c*^2^ (1, n = 35) = 1.62	> 0.10
Antidepressants	–	7 (43)	10 (52)	*c*^2^ (1, n = 35) = 0.27	>0.10
Antipsychotic agents	–	10 (62)	6 (31)	*c*^2^ (1, n = 35) = 3.34	0.06

Missing values explain the differences in degrees of freedom among tests.

BD = bipolar disorder; BD-I = bipolar I disorder; BD-II = bipolar II disorder; HAM-D = Hamilton Rating Scale for Depression; HC = healthy controls; NART = National Adult Reading Test; SD = standard deviation; YMRS = Young Mania Rating Scale.

a HC < BD-I, BD-II.

Only one subject with BD-I and five subjects with BD-II were free of drug treatment. Slightly more participants with BD-I than BD-II were taking antipsychotic drugs (p = 0.06), although among those taking these agents there were no group differences in dosage [chlorpromazine equivalents, *t*(14) = 1.04, p > 0.10]. The use of antidepressants and mood stabilizers, mostly lithium and sodium valproate, did not differ between groups. Most participants (eight BD-I and nine BD-II) were taking a combination of two drugs, with *‘antidepressants + antipsychotic agents’* or *‘antidepressants* *+ mood stabilizers’* being the most frequent combinations (n = 12). The BD groups did not differ either on the number of manic symptoms experienced during *highs* (Hypomania Checklist-32), age at first mood episode, age at first bipolar disorder diagnosis, or time from first mood episode until diagnosis of bipolarity (all p > 0.10). On average, participants in our sample were diagnosed with BD ten years after suffering their first mood episode. Approximately one-third of our sample suffered comorbid anxiety disorders, most frequently panic disorder, and slightly more individuals with BD-I than BD-II presented with a positive family history of BD, although, again, neither of those differed statistically between participants with BD-I and BD-II (Table1).

### Behavioral responses to the emotion regulation paradigm

The factor *run* had no significant effect for accuracy and only showed a significant interaction with memory load for reaction time (RT) [*F*(2,258) = 9.41, p < 0.001], indicating a decrease in RT for the 2-back condition only as the task progressed, although this effect did not differ across groups. For this reason, all the following analyses were performed collapsed across the three runs.

On average, *accuracy* [percentage of correctly identified trials (responding to targets and non-responding to fillers)] during the task was high (above 80%). A significant interaction memory load × group [*F*(2,51) = 5.43, p < 0.01] indicated the presence of group differences only during the 2-back condition, where subjects with BD-I showed lower accuracy than HC; no other pairwise comparison result was significant.

**Figure 1:**
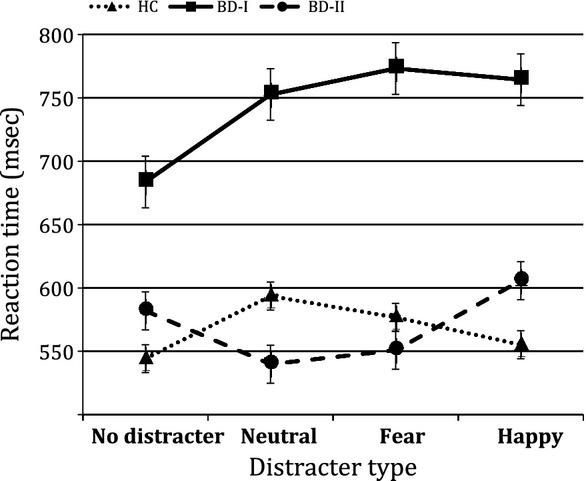
Reaction time to the 2-back targets during the presentation of different emotional distracters. Error bars correspond to the standard error of the mean. BD-I = bipolar I disorder; BD-II = bipolar II disorder; HC = healthy controls.

With regard to RT to targets, blocks in which participants responded to less than one-third of the targets were declared missing (0.8% of the total). These were distributed evenly across the groups. A significant memory load × group interaction [*F*(2,51) = 5.70, p = 0.006] indicated greater slowing in the 2-back condition relative to the 0-back for BD-I versus BD-II and HC. The interaction distracter × group was also significant [*F*(6,153) = 2.54, p = 0.022]. Due to our *a priori* hypotheses and planned analyses for the fMRI data, we only present the comparison of the effect of the distracters on RT during the demanding 2-back memory condition (the results largely overlap with the analysis collapsed across memory conditions). Pairwise comparisons across groups showed BD-I to be slower than BD-II and HC during the presence of all distracters (all p < 0.013), but no significant differences were found between BD-II and HC (Fig.1). In the absence of distracters, only BD-I differed from HC (p = 0.037). Within groups, contrasts using the no-distracter category as reference showed slowed RTs in both BD-I and HC during the presence of fear distracters (p = 0.05 and p = 0.03, respectively), but only in BD-I during the presence of happy distracters (p = 0.038). HC also showed a significant slowing during the presence of neutral distracters (p = 0.032). BD-II did not show any significant change in RT due to distracters (Fig.1).

### Blood oxygenation level-dependent (BOLD) responses to the emotional paradigm

**Figure 2:**
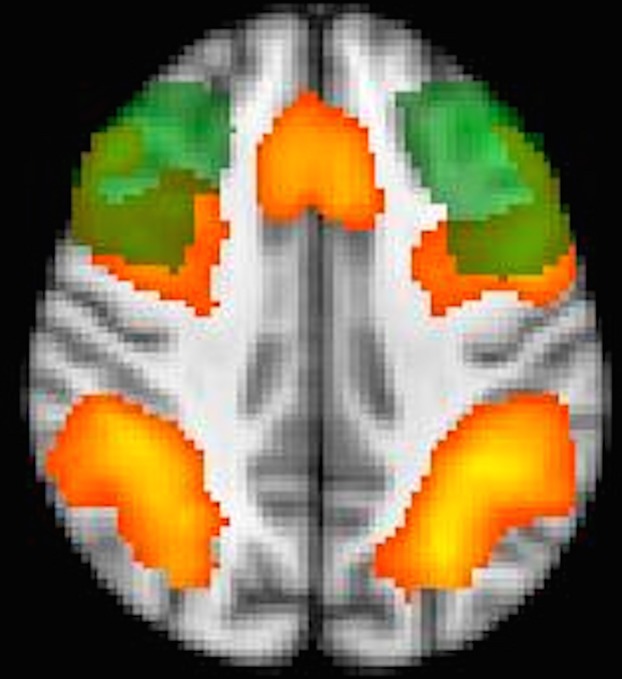
Presence of distracters. Red/yellow = increased activity in the occipito-parietal cortex, dorsolateral prefrontal cortex (DLPFC), and anterior cingulate cortex due to the presence of distracters (i.e., 2-back + any-distracters > 2-back + no-distracters). Green = anatomical mask of the middle frontal gyrus. Overlapping area = defined region of interest for the DLPFC.

Results for 2-back + no-distracters > 0-back + no-distracters showed activity within the working memory network, which was greater in BD-I versus HC and BD-II. The presence of distracters, in general (2-back + any-distracters > 2-back + no-distracters), was also associated with increased activity within the working memory network across all participants (Fig.2).

**Figure 3:**
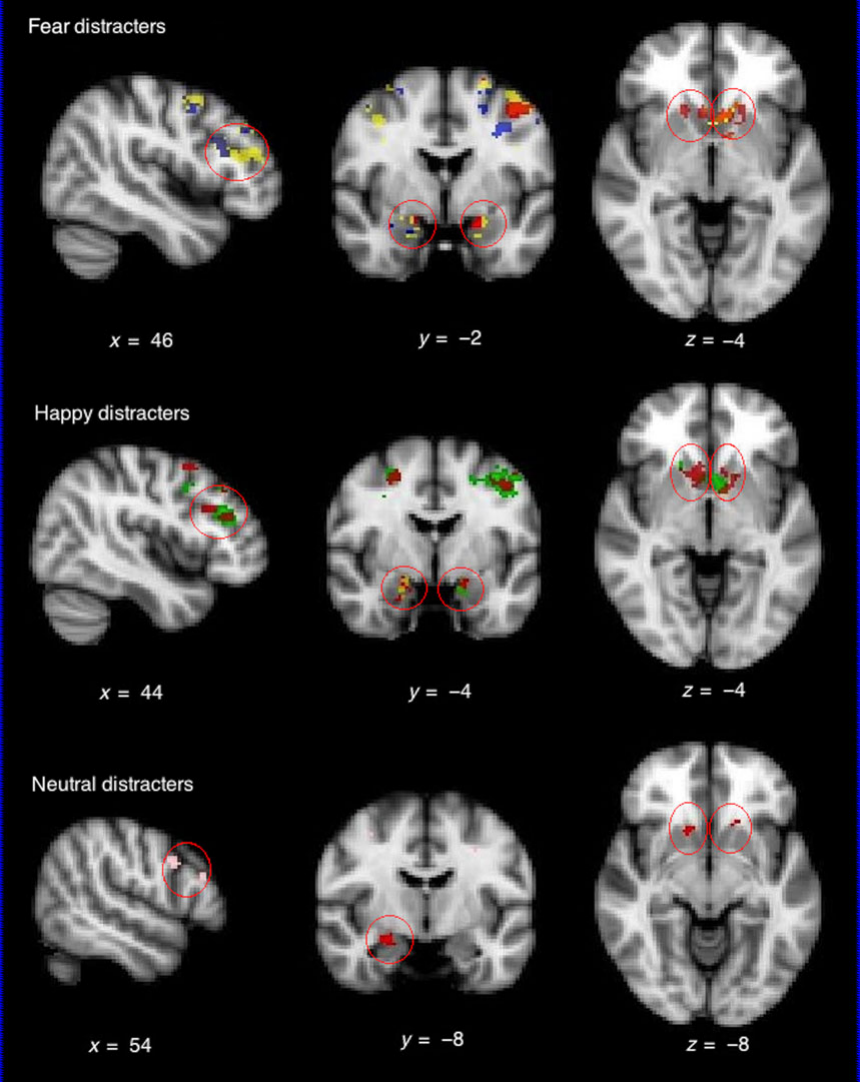
Group comparison during the presence of fear, happy, and neutral distracters during the performance of the 2-back working memory task (2-back + no-distracters memory task as baseline). Red = bipolar I disorder (BD-I) > healthy controls (HC); yellow = bipolar II disorder (BD-II) > HC; green = BD-I > BD-II; blue = BD-II > BD-I; pink = HC > BD-I. Figure shown in radiological convention (left image corresponds to right side of the brain and vice versa).

ROI analyses comparing BOLD responses across groups for each distracter type (versus no distracters) showed: (i) during the presence of fear distracters, both BD groups versus HC showed increased activity in all three ROIs, with BD-II also demonstrating increased activity versus BD-I in the DLPFC and amygdala; (ii) during the presence of happy distracters, BD-I showed increased activity in all three ROIs compared to HC and BD-II, with BD-II > HC only in the amygdala; and (iii) during the presence of neutral distracters, HC showed increased activity in the DLPFC compared to BD-I, whereas the opposite pattern appeared in the amygdala and accumbens (Fig.3).

Functional connectivity analysis (PPI) showed no group differences in the negative correlation between the DLPFC and accumbens during the presence of happy distracters. Subjects with BD-II showed a significantly greater negative correlation between the DLPFC and amygdala, bilaterally, compared to subjects with BD-I and HC during the presence of fear distracters (Table2).

**Table 2:** Mean (standard deviation) beta-weights and comparison across groups for the negative association between the time series in the dorsolateral prefrontal cortex (DLPFC) and the activity in the accumbens and amygdala (psychophysiological interaction analysis)

	HC (n = 19)	BD-I (n = 16)	BD-II (n = 19)	*F*(2,53)
**Left**				
DLPFC–amygdala	0.04 (0.14)	0.06 (0.20)	0.17 (0.15)	3.19^a^ BD-II > BD-I, HC
DLPFC–accumbens	0.07 (0.17)	0.10 (0.42)	0.11 (0.24)	0.11
**Right**				
DLPFC–amygdala	0.04 (0.13)	0.08 (0.19)	0.20 (0.23)	3.40^a^ BD-II > BD-I, HC
DLPFC–accumbens	0.05 (0.13)	0.10 (0.35)	0.09 (0.34)	0.16

BD-I = bipolar I disorder; BD-II = bipolar II disorder; HC = healthy controls.

a p < 0.05.

### Diffusion tensor imaging

There were no significant group differences in fractional anisotropy (FA) in the comparison tract or in the left uncinate fasciculus [*F*(2,49) = 0.56, p > 0.10]. However, results showed significant group differences in FA in the right uncinate fasciculus [*F*(2,52) = 4.09, p = 0.023], with BD-I showing reduced FA compared to BD-II and HC. To better interpret the potential causes of this difference, we compared longitudinal and radial diffusivity (RD) within this tract across groups. A trend effect of group was observed [*F*(2,52) = 2.84, p = 0.06] for RD. Pairwise comparisons revealed that BD-I had increased RD compared to HC (p = 0.02) and BD-II (p = 0.09).

### Effects of antipsychotic agents on previous results

Although there were no significant differences in the class or load of medication between the bipolar disorder groups, but only a tendency towards significance for antipsychotic drugs, we repeated the previous analyses including chlorpromazine equivalents as a covariate. Regarding RT, the memory load × group interaction remained significant, whereas the distracter × group effect dropped to p = 0.08. All reported positive results regarding fMRI data, and differences in FA in the right uncinate fasciculus remained significant, whereas RD group differences were no longer significant (See *Supplementary material*).

### Additional exploratory correlations across emotion regulation measures

Finally, with a purely exploratory aim, we tested whether the magnitude of the functional connectivity between the DLPFC and amygdala, as measured by PPI, was correlated with FA or RD in the uncinate fasciculi. For the entire sample, both FA and RD in the left uncinate fasciculus, but not the right, were correlated with the left DLPFC–amygdala inverse functional connectivity index (FA: *r* = 0.33, p = 0.02; RD: *r* = −0.37, p = 0.01). RD in the left uncinate fasciculus also correlated with the right DLPFC–amygdala functional connectivity (*r* = −0.35, p = 0.01). The left DLPFC–amygdala functional connectivity index also showed a subthreshold correlation (*r* = −0.24, p = 0.08) with a change in RT, caused by the presence of fear distracters (2-back + fear-distracters RT – 2-back + no-distracters RT) – that is, the greater the inverse functional connectivity, the smaller the effect of fear distracters on RT.

## Discussion

Our main goal was to compare function and white matter microstructural organization within the emotion regulation brain circuitry between euthymic BD-I and BD-II, and HC. Overall, our results were consistent with previous findings and theories of BD at pointing towards abnormalities in BD-I compared to BD-II and HC. Euthymic subjects with BD-I showed not only increased responses in the DLPFC, amygdala, and accumbens during the presence of fear and happy distracters, and, importantly, lower functional connectivity between the DLPFC and amygdala during the presence of fear distracters, but also poorer behavioral performance (slowed RT). All of the previous is indicative of an inefficient regulation of their subcortical responses to emotional distracters. In subjects with BD-I, lower FA and higher RD were also found in the white matter of the right uncinate fasciculus, which may be a core biological substrate underpinning the deficient emotion regulation seen in this group. Interestingly, euthymic participants with BD-II also showed increased BOLD responses in the DLPFC and amygdala, but, importantly, also greater inverse DLPFC–amygdala functional connectivity, during the presence of fear distracters. This group also showed intact white matter microstructure in the uncinate fasciculus, and no RT slowing during the presence of fear distracters. These findings suggest increased emotion reactivity in euthymic BD-II, as was the case for BD-I; however, unlike in the latter group, BD-II behavioral, functional connectivity and white matter microstructure results suggested no deficits in emotion regulation.

### General working memory effects

Euthymic subjects with BD-I showed a general slowing in RT when responding to targets during the 2-back condition compared to subjects with BD-II and HC, suggesting a general working memory deficit, which is consistent with the broad cognitive impairment reported in BD 30,31. This group also showed increased activity in prefrontal and parietal ‘working memory’ brain regions while performing the 2-back versus 0-back in the absence of distracters. This can be interpreted as a higher recruitment of cognitive resources in order to perform this demanding task. While interpretations of increased and decreased activity in patient groups can be problematic, this finding at least argues against the possibility that the slower RT in subjects with BD-I resulted from poor task engagement.

Differences in cognitive function between BD-I and BD-II are not reported consistently. Most studies indicate more pronounced deficits in BD-I 32–34, but some have failed to find any group differences 35 or even any impairment in BD-II 36. Consistent with the latter, in the present study euthymic subjects with BD-II performed at a very similar level to HC.

### Emotion regulation effects on behavioral and BOLD responses

During the 2-back working memory test, euthymic participants with BD-I showed a greater RT slowing during the presence of both fear and happy distracters compared to HC and BD-II, whereas subjects with BD-II did not show any slowing. It could therefore be suggested that emotional distracters had a lower impact on euthymic subjects with BD-II compared to subjects with BD-I. However, this ‘reduced impact’ was not apparent for basic emotional responses as those with BD-II showed the largest BOLD responses in the amygdala and DLPFC during the presence of fear distracters, suggesting a heightened sensitivity 4,7–10,37. There was no clear pattern of lateralized responses to emotional distracters, and most activations were present bilaterally. More importantly, BD-II showed a stronger inverse DLPFC–amygdala coupling bilaterally to fear distracters when compared to BD-I and HC, suggesting a more efficient DLPFC downregulation of amygdala reactivity. This increased functional coupling may have contributed to the reduced impact of fear distracters on the RT of subjects with BD-II. By contrast, subjects with BD-I did not show any differences in DLPFC–amygdala functional connectivity relative to HC, but did show increased activity in these regions during the presence of fear distracters, suggesting an increased emotional reactivity that may have been inefficiently downregulated.

The presence of happy distracters only produced a significant RT slowing, along with greater DLPFC, amygdala, and accumbens activity, in BD-I relative to BD-II and HC, indicating greater emotional interference from happy distracters only in this group 38. Again, this pattern of greater activity was not accompanied by an increased DLPFC–amygdala/accumbens coupling, further suggesting inefficient neural emotion regulation in euthymic BD-I.

Together, these findings concur with previous research showing a deficient coupling between executive–regulatory prefrontal areas and emotional–reactive brain structures in BD-I 7–10. Importantly, and for the first time, we show these functional abnormalities in emotion regulation circuitry not to be present in euthymic subjects with BD-II.

### White matter microstructural organization

Due to the neuronal regions with which it interconnects, the uncinate fasciculus has been highlighted as a key white matter tract for emotion regulation 16, and previous research has shown this tract, most consistently in the right hemisphere, to be abnormal in BD-I 12–15. In accordance with our behavioral and fMRI findings, euthymic subjects with BD-I in the present study showed reduced FA in the right uncinate fasciculus compared to euthymic subjects with BD-II and HC, who did not differ from each other. This finding further supports emotion regulation circuitry abnormalities being specific to BD-I and not generalized to BD-II. Furthermore, an exploratory correlation analysis showed an association between FA and RD with the functional DLPFC–amygdala coupling across the whole sample, although this was significant only for the left uncinate fasciculus. This suggests that higher fractional anisotropy and lower RD result in a greater inverse functional coupling between these two brain regions. Importantly, we also found that greater functional inverse DLPFC–amygdala coupling leads to a reduced RT interference caused by negative distracters. Together, these findings suggest that the less compromised the white matter in the uncinate fasciculus, as in subjects with BD-II relative to subjects with BD-I, the better the functional DLPFC–amygdala coupling during emotion regulation, resulting in better task performance.

Finally, there are some caveats that should be noted. Despite recruiting a well-characterized patient sample, it was only moderately sized, and some of the effects were of marginal significance, so further replication is required to confirm their validity. However, it is also important to stress that we presented converging evidence for potential deficits in emotion regulation in euthymic BD-I, but not in euthymic BD-II, from the analysis of three different response modalities: behavioral, neurofunctional, and neuroanatomical. This concordance in the results of our multimodal approach strengthens the validity of our conclusions. A second limitation of the present study lies in its cross-sectional nature, which precludes any inference of causality from our results or of stability in the processes we investigated. For example, we cannot determine whether the reduction in functional DLPFC–amygdala coupling arises owing to alterations in the white matter microstructure, whether the microstructure alters in response to reduced ‘signal handling’ load, or whether both mechanisms are concurrent but independent. Similarly, we cannot determine whether the group differences we found would also be present during mood episodes, or even whether they would remain stable throughout the course of the disease. A previous study using the same paradigm in depressed patients with BD-I 39 failed to report any abnormal activity or connectivity between the prefrontal cortices and amygdala during the presentation of fearful faces, which would suggest that this failure of emotion regulation is present only during euthymia. However, more recently, Radaelli et al. 40 and Vizueta et al. 41, both using the same face-matching paradigm, have shown deficits in connectivity between the DLPFC and amygdala in currently depressed participants with BD-I and BD-II, respectively. In light of the present and previous findings, further research on the stability of these potential deficits and their characterization during the different phases of the disease is required. Within our sample, and any BD-II sample, there is also a risk of some subjects with BD-II converting to BD-I after subsequently experiencing a full manic episode, although the fact that our BD-II sample had a mean age of around 40 years, with a long clinical history during which several hypomanic, but never manic, episodes had been experienced, makes us confident that our BDII patient sample was well established and potentially highly stable. Finally, we should also acknowledge the potential confounding effects of variables such as alcohol/drug abuse or medication. We explicitly excluded volunteers with an alcohol/drug dependence history more recent than one year prior to inclusion in the present study, and, in fact, only one participant had a past history of such problems. Moreover, our clinical groups did not differ significantly in type or load of medication, and when controlling for the potential effects of antipsychotic agents – the only drug class with a statistical trend to differ between the bipolar disorder groups – the results remained mostly unchanged. However, these factors can have a complex effect on the results that we might not have been able to control – for example, the effects of different drug combinations and the length of treatment. It is also important to note that recent reviews 42,43 suggest that drug effects have little impact on BOLD and DTI measures and that, if there were an effect, it would be in the direction of normalizing these measures relative to non-clinical populations. Therefore, we are relatively confident that our results are unlikely to be explained by medication.

In summary, euthymic patients with BD-I showed a clear interference of emotional distracters, especially the fear distracter, in their behavioral responses, along with abnormally increased activity in the amygdala and DLPFC, but no increased coupling between these brain regions, suggesting a potential neural mechanism for impaired emotion regulation. This abnormality may be associated with a compromised white matter microstructure in the right uncinate fasciculus, where patients with BD-I also showed differences relative to HC and BD-II. By contrast, while euthymic subjects with BD-II also showed increased DLPFC and amygdala activity to fear distracters, they displayed greater DLPFC–amygdala coupling, a lack of behavioral response interference, and an absence of alteration in white matter microstructure organization in the uncinate fasciculi, results suggestive of a well-preserved emotion regulation ability. Overall, our findings are consistent with recent research evidence and theoretical accounts of BD which emphasize deficient emotion regulation in the pathophysiology of the disorder 3,4, but suggest important differences between BD-I and BD-II in this process.

## Supporting Information

**Data S1.** Extended methods and control for medication effects.
