# Supplementary material for: Emotion regulation deficits in euthymic bipolar I versus bipolar II disorder: a functional and diffusion-tensor imaging study
Source: Bipolar Disord. 2015 Mar 15;17(5):461–70. doi: 10.1111/bdi.12292 (PMC4672703; doi:10.1111/bdi.12292)
Supplement: Supplementary file 1 — Data S1. Extended methods and control for medication effects. [file bdi0017-0461-sd1.docx]

**Supplementary material**

**Experimental paradigm**

The experimental paradigm used here replicated that implemented by Mullin *et al^1^*. Participants were presented with a random stream of letters from which they had to identify, through button press, either the letter M (0-Back, control condition) or 2-Back repetitions (A-B-A type, working memory condition). Each of these memory load conditions (0-, 2-Back) was presented four times in combination with the ‘distracters’ variable (no-distracters, neutral, fearful or happy facial expressions). Each of the resulting 8 memory load x distracter conditions was presented in blocks including 12 trials each with 5 of these trials containing a target (either an M or a 2-Back repetition depending on the memory condition). Blocks were randomly presented across participants with the only constraint being that the first block was always 0-Back+No-distracters. The whole task was run 3 times for each participant.

Letter stimuli were presented for 500 milliseconds, with a jittered ITI of mean 3,500 milliseconds. Facial stimuli were grayscale male and female pictures from the NimStim facial expression series^2^. Two identical faces flanked the letter stimuli on each trial, always located at the same spatial location across trials (Supplementary Figure 1). Distracters changed almost simultaneously with the appearance of the new letter.

***Supplementary Figure 1.*** *Example of the 2-Back Happy-distracters condition. The second ‘G’ letter represents a target.*

**MRI data acquisition**

During each run of the task 212 brain volumes of 35 slices (3.2mm thick and AC-PC orientated) were acquired interleaved with no gap through Gradient-echo echoplanar images using a 3T General Electric HDx MRI Scanner (TR=2s, TE=35msec, flip angle=90^0^, FOV=205mm, in-plane resolution=64x64, voxel size=3.2mm isotropic). **Voxel size after normalization into MNI space was 2mm isotropic**. Each run started with 6 dummy scans to set longitudinal magnetization into steady state.

For co-registration of fMRI data into standard space a 3D FSPGR image was obtained for each participant (TR= 7.9msec, TE= 3.0msec, inversion time=450msec, flip angle= 20^0^, acquisition matrix= 256(AP) x 192(LR) x 172(SI), 1mm isotropic voxels). Registration of fMRI data was optimized using high-resolution field-maps.

Diffusion-weighted MR data (DTI) were acquired with a twice-refocused spin-echo echo-planar imaging sequence providing parallel AC-PC brain coverage. Acquisition was peripherally gated to the cardiac cycle. Data were acquired from 60 slices of 2.4 mm thickness, FOV= 230mm, matrix size 96×96, TE= 87ms and parallel imaging (ASSET factor = 2), b-value= 1200 s/mm^2^, encoding diffusion along 30 isotropically distributed directions and three non-diffusion-weighted scans according to an optimized gradient vector scheme^3^.

**fMRI data pre-processing**

fMRI data were pre-processed using the following steps: motion correction using MCFLIRT^4^; non-brain removal using BET^5^; spatial smoothing using a Gaussian kernel of FWHM 5mm; grand-mean intensity normalization of the entire 4D dataset; highpass temporal filtering of frequencies>0.02Hz; and registration to individual T1 anatomical images using FLIRT^6^. No participant was excluded due to excessive head movement since none presented >3mm displacements in any direction. Also, head movement parameters did not differ between groups, suggesting that group differences could not be due to differences in head movement.

The task was modeled within the GLM framework and one regressor for each condition (memory x distracter type) was defined except for the 0-back+no-distracters, which constituted the baseline. Based on our a-priori hypotheses, we defined specific contrast to test each of those. In order to investigate the main effect of distracters, we defined the contrast 2-back+any-distracter(fear, happy, neutral) > 2-back+no-distracters. However, to further investigate the distracter-specific effects, we also included the specific contrasts 2-back+(fear|happy|neutral)-distracters > 2-back+no-distracters. General activation within the working memory network was examined through the contrast 2-back+no-distracters > 0-back+no-distracters.

**Drawing of Regions-of-Interest (ROI)**

The ROIs for the amygdala and accumbens were standard ROIs from the Harvard-Oxford structural atlas provided in FSL. For the DLPFC ROI and due to the lack of a clear anatomical definition of DLPFC, a combined functional/structural ROI was created as follows: The binarized mean activity map across all participants resulting from the contrast 2-back+any-distracter > 2-back+no-distracter was multiplied by a binarized anatomical map of the middle frontal gyrus obtained from the Harvard-Oxford cortical structural atlas. This strategy resulted in a region within the middle frontal gyrus that was activated by the presence of distracters during the cognitively demanding 2-back condition relative to the absence of distracters under the same cognitive demands.

**DTI data pre-processing and extraction**

Using the software package ExploreDTI^7^, the DTI images were corrected for distortions introduced by the diffusion-weighting gradients and for between-slice motion with appropriate reorienting of the encoding vectors^8^ and modulation of signal intensity^9^ before a tensor model^10^ was fit to the data using a RESTORE – a robust estimation routine that rejects outlier data during the regression to the tensor model^11^. Head movement parameters did not differ across groups, suggesting that group differences could not be influenced by differences in head movement. Deterministic fiber tracking was initiated from the center of every voxel in the entire brain where the fractional anisotropy exceeded a threshold of 0.15. A fourth-order Runge-Kutta solution was used to evolve a space-curve making step-sizes of 0.5 mm and continuing subject to a termination criterion of fractional anisotropy (FA) > 0.15 or a turning angle exceeding 60°. At the completion of ‘whole brain tractography’ in this way, the uncinate fasciculi and the cortical spinal tract – lower section -were then virtually dissected by manually drawing, in native space for each individual, regions of interest (ROIs) to act as AND ‘gates’ that the tract must pass through or NOT ‘gates’ that it should not pass through, using landmark techniques that have previously been shown to be highly reproducible^12^. For the uncinate fasciculus, an AND gate was drawn on a coronal slice around the region where the uncinate enters the frontal lobe immediately rostral to the genu of the corpus callosum (Supplementary Figure 2). A second AND gate was drawn on an axial slice capturing the uncinate bundle at the point where it bends into the inferior temporal lobe region. This bend was visually identified on the midline sagittal plane with the axial slice being placed at a level immediately dorsal to the upper pons. A NOT gate was drawn across the coronal slice level with the front of the pons to remove tracts of the internal frontal-occipital fasciculus. The tract was then visually inspected and any obvious outlier streamlines that were not consistent with the known uncinate fasciculus anatomy^13,14^ were removed using additional NOT gates. This procedure was performed for both hemispheres separately. For the cortical spinal tract we used the same methodology than previously described by Keedwell *et al^15^.*

The operator drawing these ROIs was blind to the participants' group allocation. The mean fractional anisotropy, mean diffusivity(MD), longitudinal diffusivity and radial diffusivity were then calculated for the reconstructed pathways in ExploreDTI by averaging the values sampled at each 0.5 mm step along the pathways^16^, providing tract-specific means for the left and the right hemispheres. A second blind operator independently obtained FA and MD values for the left and right uncinate fasciculi using the same method in a subset of 12 randomly selected participants. The resulting inter-rater reliability was very high (smallest r = 0.93, p< .0001).


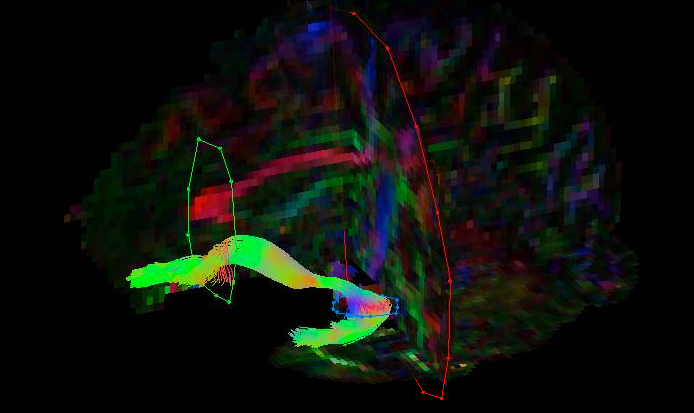


***Supplementary Figure 2.*** Visual representation of the reconstructed left uncinate fasciculus of one participant. The AND gates used for tracking the uncinate fasciculus in this case are shown in green and blue. The NOT gate is shown in red.

**Effects of antipsychotic drugs on positive results**

Our sample included a slightly larger number of participants with BD-I taking antipsychotic drugs compared to BD-II. There have been reports suggesting that this class of drug might affect neuroimaging measures, although reported effects have mostly shown a tendency towards normalization^17,18^. In any case, we repeated our analyses including a chlorpromazine equivalent transformation (coded 0 for those not taking this class of medication^19^) as a covariate of no-interest. Most results remained unchanged, and only the ANOVA for Radial Diffusivity in the right uncinate fasciculus was no longer significant (supplementary table 1 and figure 3).

Since antipsychotics have clearly shown to slow down reaction time, we also ran Student’s t-test analyses for this behavioral output reproducing the pairwise comparisons carried out within the main ANOVA, but only including those patients not taking antipsychotics (n=6 BD-I and 13 BD-II). The results still showed significantly reduced reaction time in BD-I during neutral and fear distracters, but not anymore for happy distracters. As before, there were no group differences for the no-distracters condition.

***Supplementary table 1.*** Main results from the analyses including Chlorpromazine equivalents as a covariate of no-interest.

| Effect | Statistic |
| --- | --- |
| *RT*  load x group  distracter x group | F(2,50)= 3.97; p= .02  F(6,150)= 1.92; p= .08 |
| *PPI (Fear distracters)*  Right DLPFC-amygdala  Left DLPFC- amygdala | F(2,53)= 3.35; p= .04  F(2,53)=3.13; p= .05 |
| *DTI*  FA  RD | F(2,52)= 3.39; p= .04  F(2,52)= 2.22; p= .11 |

RT: Reaction Time; PPI: Psychophysiological Interaction analysis; DTI-FA: Diffusion Tension Imaging derived Fractional Anisotropy, DTI-RD: Diffusion Tension Imaging derived Radial Diffusivity

***
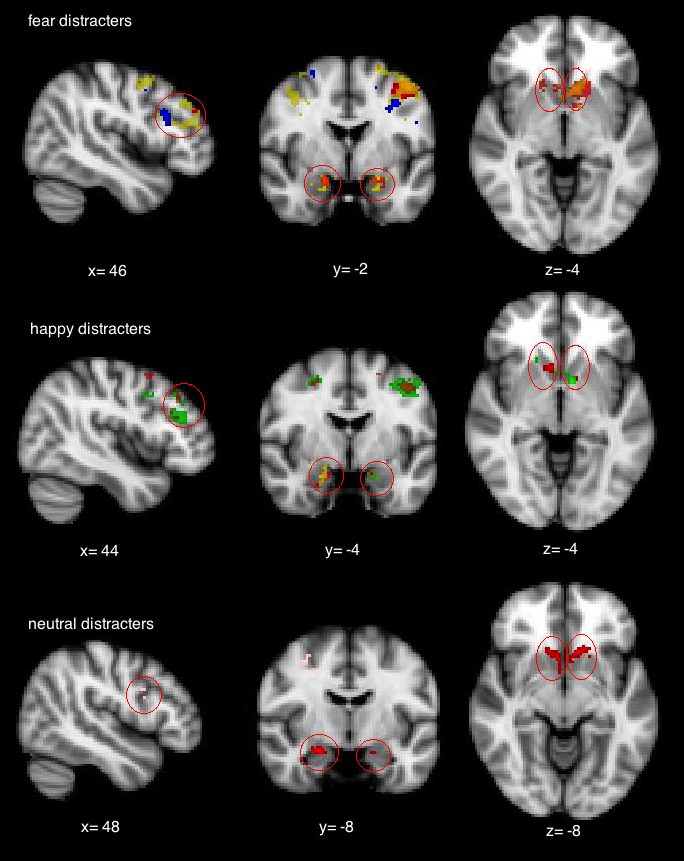
Supplementary Figure 3.*** Group comparison for fear, happy and neutral distracters during the performance of the 2-back working memory task using Chlorpromazine equivalents as covariate. BD-I>HC = Red; BD-II>HC = Yellow; BD-I>BD-II= Green; BD-II>BD-I= Blue; HC>BD-I= Pink. Figure shown in radiological convention (left image corresponds to right side of the brain and vice-versa).

**References**

**1.** Mullin BC, Perlman SB, Versace A, Almeida JRC, LaBarbara EJ, Klein C, Ladouceur CD, Phillips ML. An fMRI study of attentional control in the context of emotional distracters in euthymic adults with bipolar disorder. Psychiatry Res 2012; 201: 196-205.

**2.** Tottenham N, Tanaka JW, Leon AC, McCarry T, Nurse M, Hare TA, Marcus DJ, Westerlund A, Casey BJ, Nelson C. The NimStim set of facial expressions: judgments from untrained research participants. Psychiatry Res 2009; 168: 242-249.

**3.** Jones DK, Horsfield MA, Simmons A. Optimal strategies for measuring diffusion in anisotropic systems by magnetic resonance imaging. Magn Reson Med 1999; 42: 515-525.

**4.** Jenkinson M, Bannister P, Brady M, Smith S. Improved optimisation for the robust and accurate linear registration and motion correction of brain images. Neuroimage 2002: 17: 825-841.

**5.** Smith S. Fast Robust Automated Brain Extraction. Hum Brain Mapp 2002; 17: 143-155.

**6.** Jenkinson M, Smith S. A global optimization method for robust affine registration of brain images. Med Image Anal 2001; 5: 143-156.

**7.** Leemans A, Jeurissen B, Siibers J, Jones DK. ExploreDTI: a graphical tool box for processing, analyzing, and visualizing diffusion MR data. Paper presented at 17th Anuual Meeting of the International Society of Magnetic Resonance in Medicine (Hawaii); 2009.

**8.** Leemans A, Jones DK. The B-matrix must be rotated when correcting for subject motion in DTI data. Magn Reson Med 2009; 61: 1336-1349.

**9.** Jones DK. Precision and accuracy in diffusion tensor magnetic resonance imaging. Top Magn Reson Imaging 2010; 21: 87-99.

**10.** Basser PJ, Mattiello J, LeBihan D. Estimation of the effective self-diffusion tensor from the NMR spin echo. J Magn Reson 1994; 103: 247–254.

**11.** Chang LC, Jones DK, Pierpaoli C. RESTORE: robust estimation of tensors by outlier rejection. Magn Reson Med 2005; 53: 1088-1095.

**12.** Catani M, Howard RJ, Pajevic S, Jones DK. Virtual in vivo interactive dissection of white matter fasciculi in the human brain. Neuroimage 2002; 17: 77–94.

**13.** Crosby, Humphrew T, Lauer EW. Correlative anatomy of the nervous system. NewYork: Macmillian, 1962.

**14.** Schmahmann JD, Pandya DN. Fiber pathways of the brain. NewYork: Oxford UP, 2006.

**15.** Keedwell PA, Chapman R, Christiansen K, Jones DK. Cingulum white matter in young females at risk of depression: the effect of family history and anhedonia. Biol Psychiatry 2012; 72: 296-302.

**16.** Jones DK, Catani M, Pierpaoli C, Reeves SJ, Shergill SS, O'Sullivan M, Maguire P, Horsfield MA, Simmons A, Williams SC, Howard RJ. A diffusion tensor magnetic resonance imaging study of frontal cortex connections in very late-onset schizophrenia-like psychosis. Am J Geriatr Psychiatry 2005; 13: 1092-1099.

**17.** Hafeman DM, Chang KD, Garrett AS, Sanders EM, Phillips ML. Effects of medication on neuroimaging findings in bipolar disorder: an updated review. Bipolar Disord 2012; 14: 375-410.

**18.** Phillips ML, Travis MJ, Fagiolini A, Kupfer DJ. Medication effects in neuroimaging studies of bipolar disorders. Am J Psychiatry 2008; 165: 313-320

**19.** Almeida JR, Versace A, Hassel S, Kupfer DJ, Phillips ML. Elevated amygdala activity to sad facial expressions: a state marker of bipolar but not unipolar depression. Biol Psychiatry 2012; 67: 414-421.
